# *In vitro* studies on space-conforming self-assembling silk hydrogels as a mesenchymal stem cell-support matrix suitable for minimally invasive brain application

**DOI:** 10.1038/s41598-018-31905-5

**Published:** 2018-09-12

**Authors:** I. Osama, N. Gorenkova, C. M. McKittrick, T. Wongpinyochit, A. Goudie, F. P. Seib, H. V. O. Carswell

**Affiliations:** 1 Strathclyde Institute of Pharmacy and Biomedical Sciences, University of Strathclyde, Glasgow, UK; 2 Leibniz Institute of Polymer Research Dresden, Max Bergmann Center of Biomaterials Dresden, Hohe Strasse 6, 01069 Dresden, Germany

## Abstract

Advanced cell therapies require robust delivery materials and silk is a promising contender with a long clinical track record. Our aim was to optimise self-assembling silk hydrogels as a mesenchymal stem cell (MSC)-support matrix that would allow future minimally invasive brain application. We used sonication energy to programme the transition of silk (1–5% w/v) secondary structure from a random coil to a stable β-sheet configuration. This allowed fine tuning of self-assembling silk hydrogels to achieve space conformity in the absence of any silk hydrogel swelling and to support uniform cell distribution as well as cell viability. Embedded cells underwent significant proliferation over 14 days *in vitro*, with the best proliferation achieved with 2% w/v hydrogels. Embedded MSCs showed significantly better viability *in vitro* after injection through a 30G needle when the gels were in the pre-gelled versus post-gelled state. Silk hydrogels (4% w/v) with physical characteristics matching brain tissue were visualised in preliminary *in vivo* experiments to exhibit good space conformity in an ischemic cavity (intraluminal thread middle cerebral artery occlusion model) in adult male Sprague-Dawley rats (n = 3). This study informs on optimal MSC-hydrogel matrix conditions for minimally invasive application as a platform for future experiments targeting brain repair.

## Introduction

Cell-based therapy, especially the use of stem cells, is one of the main approaches that successfully promotes neurorestoration of function and improves outcomes such as angiogenesis, neuroprotection, immune response and modulated inflammation in experimental models^1^. Stem cell therapy is now showing signs of success in human clinical trials^2^. For example, mesenchymal stem cells (MSCs) are appealing because these cells can be derived from an autologous cell source and they promote the repair of neurovascular units and exert potent immunomodulatory functions that result in neurorestorative effects in experimental models^3^. However, one key issue hindering the translation of cell therapies to the clinic is the lack of suitable cell delivery technologies that can support cell survival and provide transplanted cells with the necessary cues to perform their intended restorative functions such as trophic factor production^3,4^.

These support and cueing functions are normally provided by the extracellular matrix (ECM), which is critical for the storage and presentation of growth and signalling factors and the provision of cell adhesion sites to guide and promote proliferation and survival. Physical cues, such as substrate elasticity and surface topography, also direct stem cell lineage commitment^5,6^. Therefore, the ECM represents a critical signalling hub that allows (stem) cells to respond to their environment. The ECM can be mimicked quite effectively by engineered hydrogels^7,8^; however, clinical translation of stem cell therapies still requires the development of engineered materials to support function. Ideally, hydrogel materials should be biocompatible, biodegradable, minimally invasive, programmable (i.e. solution-gel transition), cytocompatible and space conforming, and they should match brain tissue mechanics, show no material swelling and support even cell distribution (reviewed in^9,10^).

Many hydrogels presently being explored as carriers for cell therapies^9,10^ are derived from a broad spectrum of materials, including tissue-derived extracellular matrix, synthetic polymers, biopolymers, peptides and hybrid materials (reviewed in^11,12^). Examples include heparin^13^ or hyaluronic acid^14^ polyethylene glycol hybrid hydrogels, self-assembling RADA (ref.^15^) peptides functionalised with a laminin-derived IKVAV motif^16^, alginate hydrogels^15^ and self-healing chitosan hydrogels^17^. Other materials include extracted extracellular matrix from Engelbreth-Holm-Swarm mouse sarcoma (i.e. Matrigel) or collagen from bovine or rodent tissues^18^. More recently, basement membrane and tunica propria ECM isolated from porcine urinary bladder is emerging as an interesting alternative to existing ECM preparations^19,20^.

All these systems have shown promise for brain repair, but these materials continue to show limitations. Emerging evidence suggests that ECM tissue specificity is important for obtaining the desired treatment outcome^21^, so ECM extracts from tumours or tissues that are unrelated to the brain are likely to require further optimisation. Single ECM component systems, such as collagen-based hydrogels, have been used extensively (reviewed in^11^), but these hydrogels have poor mechanical properties, limited resistance to biodegradation (or ability to fine tune this) and a marked tissue response^22^. Collagen is also predominantly of bovine origin, so its use in the nervous system raises theoretical risks of introducing prions directly into the brain. The development of biomimetic hydrogels (e.g. peptides, peptoids) and biohybrid hydrogel systems is promising, although these systems still require significant research efforts to yield self-assembling hydrogels with sufficiently robust mechanical properties, *in vivo* biocompatibility and the desired performance^12,23^.

The silk from *Bombyx mori* has a long track record of use in humans^24^, as this biopolymer is clinically approved as a suture and surgical mesh^25^. Its renowned mechanical properties^26^, biocompatibility and biodegradability^27^ make silk an ideal contender for applications that go beyond its current clinically approved load-bearing applications. The silk fibre can be completely reverse engineered into an aqueous silk solution^28^, which can then be processed into many different formats, including physically cross-linked hydrogels. It also has the ability to self-assemble in response to environmental triggers, making it particularly appealing for cell delivery applications (reviewed in^29^). Furthermore, silk is able to support cell growth and cell differentiation of various stem cells, including pluripotent^30^, neural^31,32^ and MSC types^33^. Silk has therefore been investigated in a range of regenerative medicine applications; however, at present, no studies have optimised self-assembling silk hydrogels-MSC conditions as a platform for future experiments on cell therapy into the brain.

Towards that, we hypothesized that *B. mori* silk could be used to generate a delivery system suitable for (i) minimally invasive application (for example, stereotactic injection for intracerebral delivery) and (ii) MSC support. For minimally invasive brain administration and stem cell support, the solution-gel characteristics and substrate elasticity are critical so we therefore focussed on optimising the physical and chemical cues of self-assembling silk hydrogels for brain administration and stem cell support. Therefore the aim of the present study was to fine-tune the processing parameters of self-assembling silk hydrogels to generate a delivery system that shows controllable solution-gel kinetics, elasticity, space conformity and MSC-biocompatibility appropriate for administration to the brain.

## Results

### Physical assessment of self-assembling silk hydrogels

**Figure 1:**
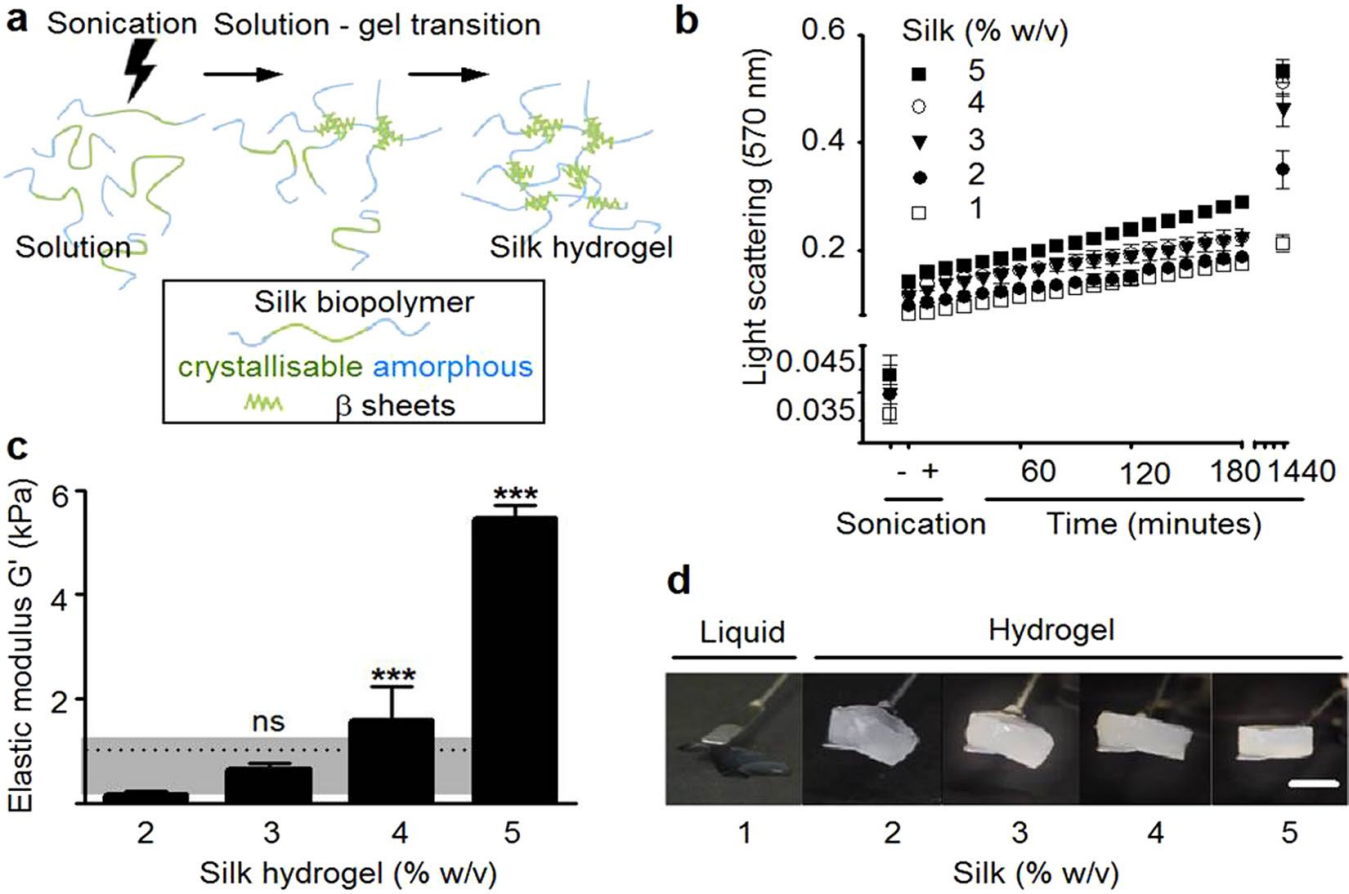
Self-assembling silk hydrogel manufacture and structural characteristics. The resulting hydrogels show controllable solution-gel kinetics and mechanical properties. (**a**) Schematic illustration of the manufacture of silk hydrogels from reverse engineered silk. (**b**) Light scattering to assess the formation of silk nanocrystallites over time. (**c**) Matrix elasticity of silk hydrogels measured by rheology (dotted line indicates typical stiffness reported for brain tissue while shading indicates the reported spectrum). (Mean ± SD, n ≥ 3 independent experiments; n.s. (non-significant) or ***P < 0.001 versus 2% w/v self-assembling silk hydrogel). (**d**) Representative macroscopic images of self-assembling silk hydrogels with increasing concentrations (scale bar 10 mm).

The solution-gel characteristics and elasticity of self-assembling silk hydrogels were assessed because control of these parameters is critical for minimally invasive applications, including brain delivery. Silk self-assembly was initiated by sonication energy (Fig. 1a) and the visible kinetics of silk nanocrystal formation was monitored by light scattering (Fig. 1b). For all silk concentrations, energy input increased light scattering even at the first measurement point (i.e. within 10 minutes of energy input) when compared to the respective untreated silk solution controls. Over the subsequent 240 minutes, light scattering increased in a linear fashion; at 24 hours, the silk hydrogels showed the highest light scattering properties over the time frame tested. The extent of light scattering depended on the amount of silk present: the highest scattering values were observed for the 5% w/v hydrogels gels and lowest for 2% w/v hydrogels (note that 1% w/v silk did not form a robust hydrogel). Increasing the amount of silk also significantly changed the rheological properties, which were used to estimate silk hydrogel elasticity (Fig. 1c). The lowest substrate elasticity was observed for 2% w/v silk hydrogels and a near exponential increase was observed, from 0.17 to 5.46 kPa, for 2 and 5% w/v silk hydrogels, respectively. Visual inspection of the silk hydrogels (Fig. 1d) supported the observations made by light scattering and rheological measurements. First, an increasing level of opaqueness (i.e. light scattering) was evident for 1 to 5% w/v silk samples (note that the liquid 1% w/v silk sample was also opaque). Second, the overall mechanical strength increased for the 2 to 5% w/v silk hydrogels, while 1% silk remained liquid.

### Structural assessment of self-assembling silk hydrogels

**Figure 2:**
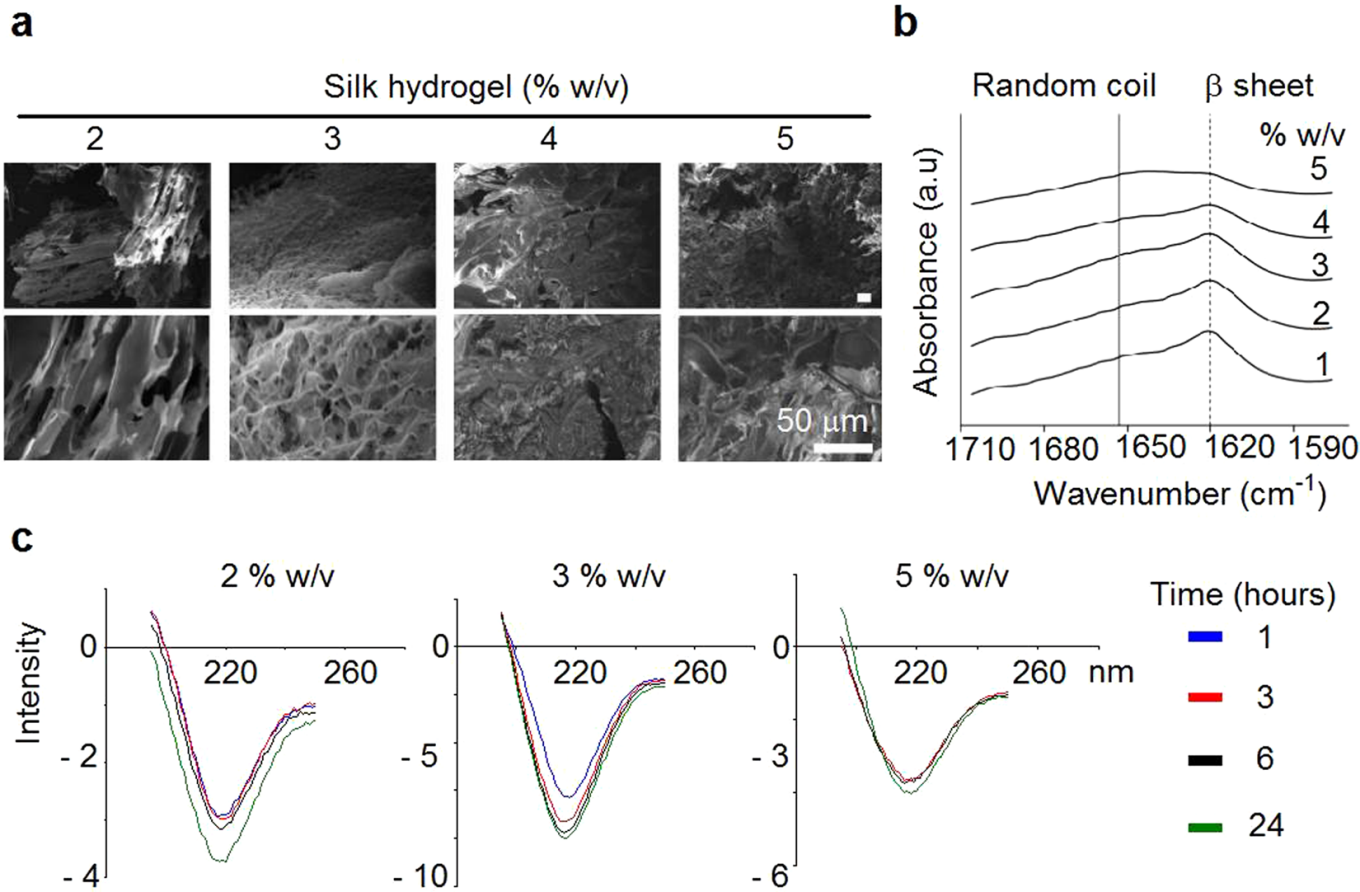
Morphology and secondary structure of self-assembling silk hydrogels. (**a**) Representative scanning electron micrographs of silk hydrogels. (**b**) FTIR spectra of silk hydrogels with absorption bands for random coils (solid line) and β-sheets (dashed line, 1625 cm^−1^) (n = 2 independent experiments). (**c**) Circular dichroism measurements of a silk solution undergoing solution-gel transition following sonication energy input (note Ellipticity at 217 nm represent β-sheets) (one independent experiment).

Scanning electron microscope of silk hydrogels showed a porous silk structure for 2% w/v silk hydrogels and progressively less porosity for the high concentration samples (Fig. 2a). Secondary structure analysis by FTIR analysis showed an amide I absorption peak at 1625 cm^−1^ (i.e. β-sheet) for all silk hydrogels (Fig. 2b). FTIR measurements were complemented by dynamic CD measurements of silk hydrogels. Ellipticity at 217 nm was used to track β-sheet conformation between 1 and 24 hours post sonication. At a lower silk content, the hydrogel showed a time-dependent increase in β-sheet content over 24 hours, whereas hydrogels with a higher silk content had essentially completed β-sheet formation by 1 hour post sonication.

### Space conformity and absence of swelling for self-assembling silk hydrogels

**Figure 3:**
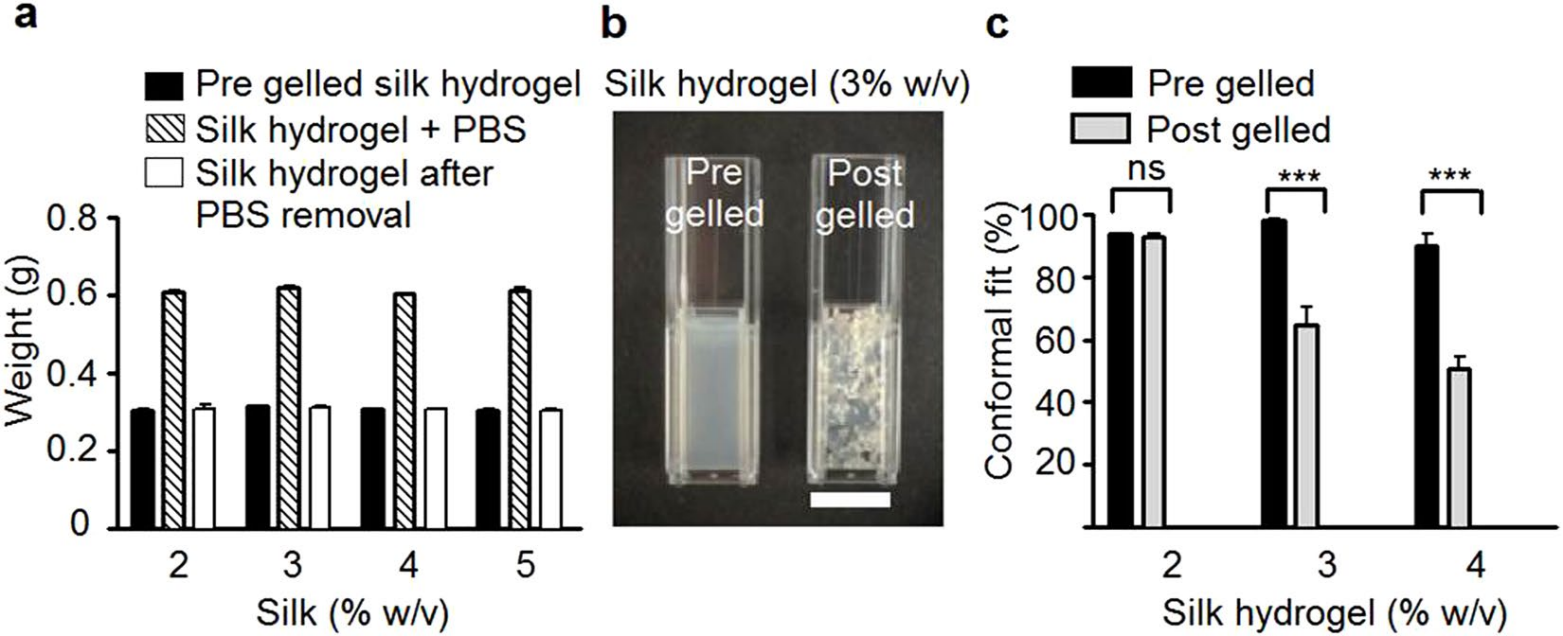
Space conformity and swelling of self-assembling silk hydrogels. (**a**) Assessment of swelling of self-assembling silk hydrogels. Weight recordings of silk undergoing the solution-gel transition; the respective hydrogel weights before, during and after incubation with phosphate buffered saline. (**b**) Qualitative images of 3% w/v silk samples added to a cuvette in the pre- and post-gelled state using a sample volume of 1 ml (scale bar 10 mm). (**c**) Quantitative assessment of space conformity of silk hydrogels. (Mean ± SD, error bars hidden in bars when not visible, n = 3 to 6 independent experiments; ***P < 0.001).

Space conforming properties without swelling are important properties as the materials designed to fill a cavity should not compress the surrounding tissue in order to be minimally invasive. The *in vitro* swelling studies showed that the weights of the self-assembling silk hydrogels did not differ during the solution-gel transition or following overnight incubation in PBS (Fig. 3a).

The conformity of the self-assembling silk hydrogels was then assessed *in vitro* before and after the completion of the solution-gel transition. Visual inspection showed that only silk samples that were still in the solution state achieved complete space filling (Fig. 3b, left cuvette). A quantitative measurement of space conformity was devised by determining the amount of silk that was able to fill a pre-defined space. For 3% w/v and 4% w/v silk samples, significantly more silk could be applied to a fixed volume when silk was in the solution state rather than in the gelled state; however, no statistical difference was observed for 2% w/v silk samples (Fig. 3c).

**Figure 4:**
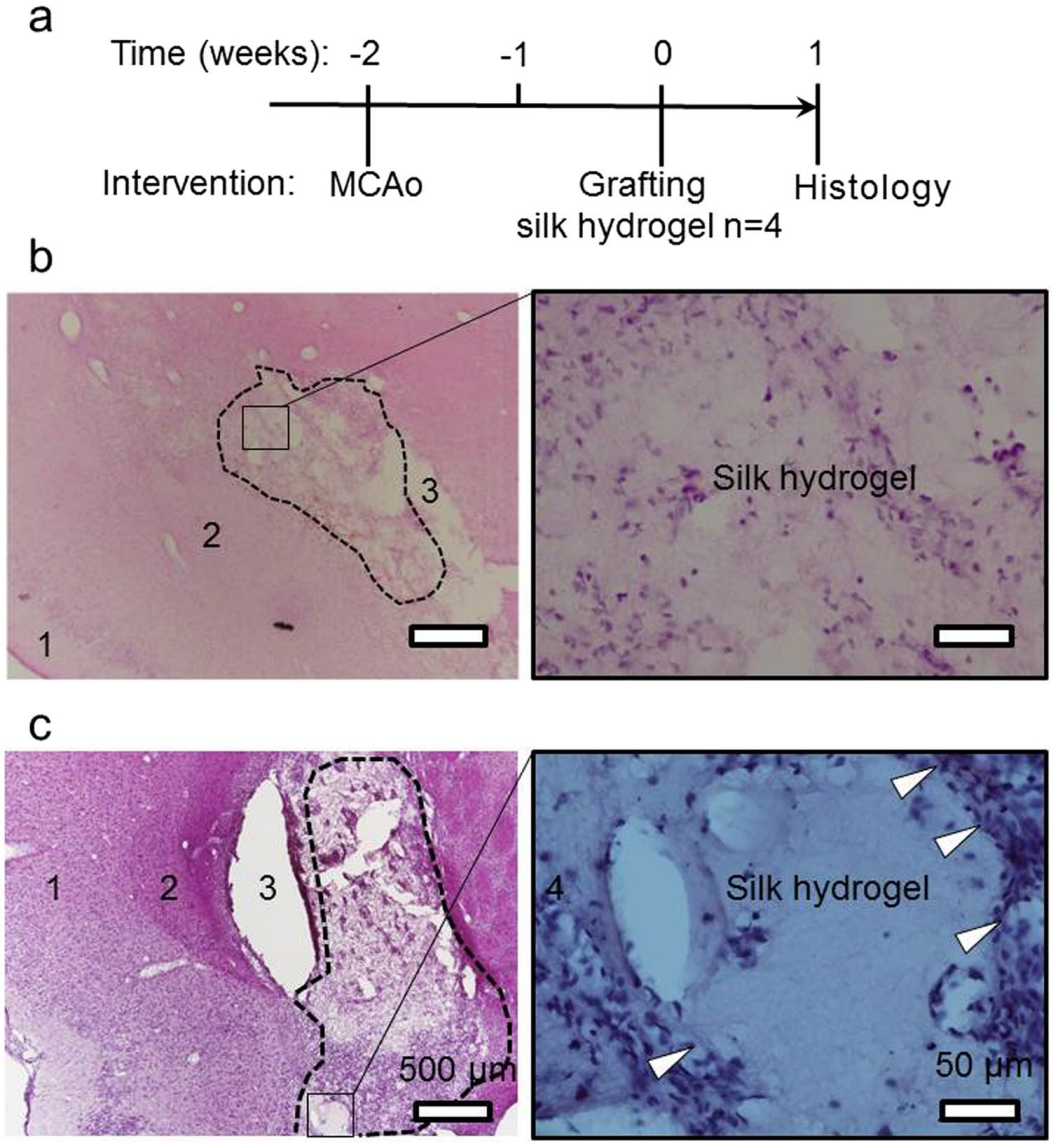
Space conformity of self-assembling silk hydrogels in lesions of variable sizes. (**a**) Experimental timeline for the stroke model in rats, injection of 4% w/v self-assembling silk hydrogel and assessment. Representative images of coronal H&E stained sections at the level of (**b**) the Bregma 1.2 mm; small-sized lesion (dotted outline), 1: cortex; 2: boarder between cortex and striatum; 3: silk graft. Boxed area and zoom show hydrogel inside the small lesion; and (**c**) at the level of the Bregma −0.26 mm; medium-sized lesion (dotted outline), 1: cortex; 2: boarder between cortex and striatum; 3: processing artefact resulting in splitting of section. Boxed area and zoom show the hydrogel area filling the cavity and surrounded by cells (arrows). See Supplementary Fig. S3 for the respective lesion topography.

Preliminary *in vivo* experiments, were designed to provide qualitative proof of concept insight to support these *in vitro* results on space conformity. One excellent model system is experimental stroke which provides a cavity for space conformity to be visualised. After chronic cerebral ischemia, application of the self-assembling silk hydrogels to the affected hemisphere resulted in excellent conformal fit (Supplementary Fig. S1). However, injection of self-assembling silk hydrogels in mice with no apparent infarct cavity (i.e. the lesion had not fully developed) resulted in silk hydrogel accumulation in the ventricle with excellent space conformity (Supplementary Fig. S1). In rats with small and medium lesions (i.e. cavities of variable sizes, detailed in Supplementary Fig. S3), injection of self-assembling silk hydrogels into the affected area showed excellent space conformity, a seamless tissue-silk hydrogel interface, and no gross adverse tissue response 7 days after application (Fig. 4). The silk hydrogel appeared intact, with no obvious signs of degradation.

### Cytocompatibility, injectability and distribution of MSCs in self-assembling silk hydrogels

**Figure 5:**
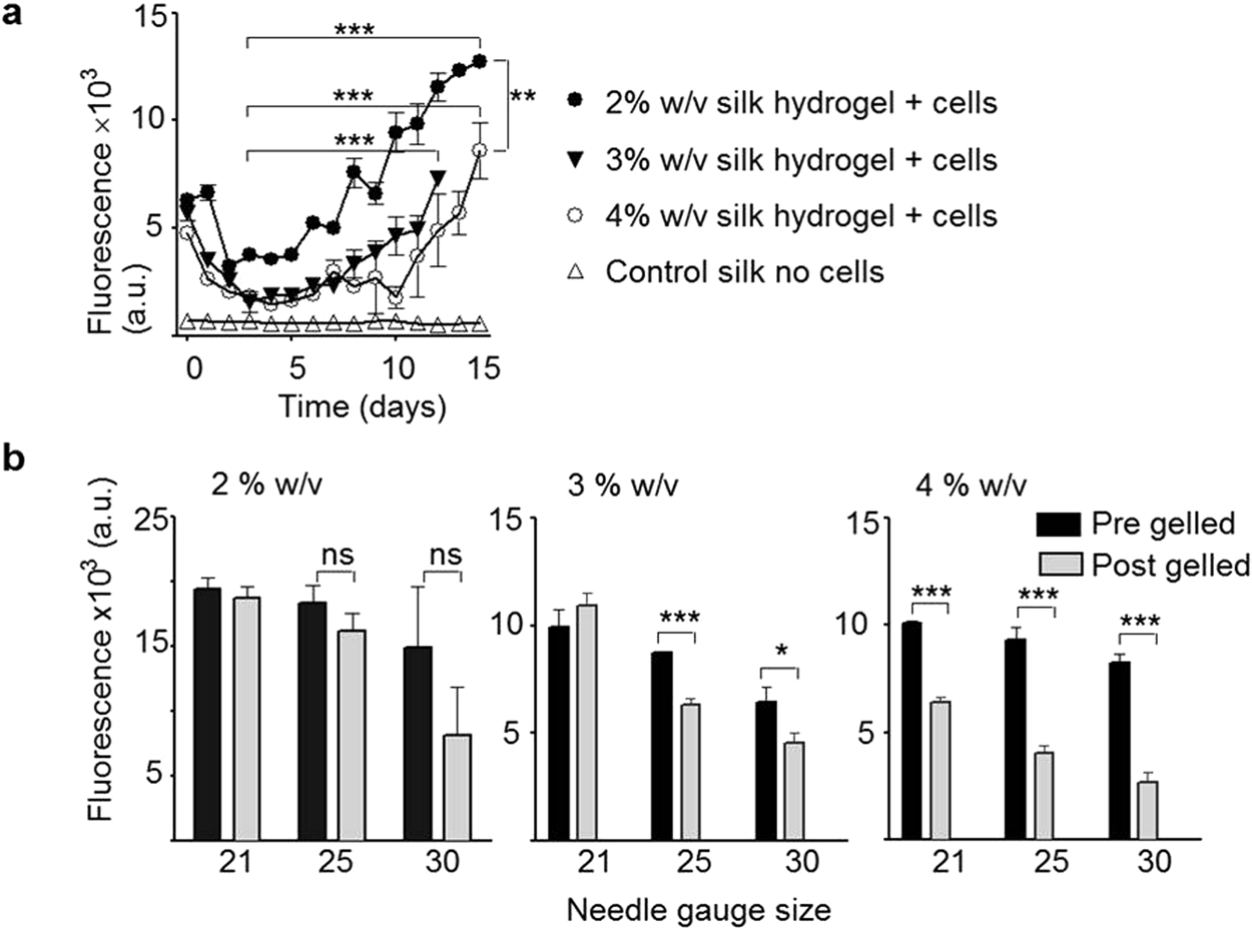
Cytocompatibility of MSCs in self-assembling silk hydrogels. (**a**) Viability of MSCs encapsulated in self-assembling silk hydrogels for up to 14 days. (**b**) Viability of MSCs encapsulated in pre-gelled or post-gelled silk hydrogels and injected through 21, 25 and 30 gauge needles. (mean ± SD, n ≥ 3 independent experiments; *P < 0.05, **P < 0.01, ***P < 0.001).

Exploiting self-assembling silk hydrogels for cell delivery requires that these systems be cytocompatible, injectable and tuneable to control cell distribution within the three dimensional hydrogel structure. An initial drop in MSC viability was observed for all hydrogels (Fig. 5a). For 2% w/v silk hydrogels, the cell viability was stabilised at day 2 and significant cell proliferation was observed from day 5 onwards, over the next 10 days, until the end of the study period. Of all the hydrogels studied, the 2% w/v hydrogels supported the best MSC growth (Fig. 5a). For 3% w/v and 4% w/v hydrogels, the cell viability stabilised at day 3 and then steadily increased over the remaining culture period (up to 14 days) (Fig. 5a).

The impact of injection shearing on cell viability was also assessed using silk still in the solution state and in the gelled state (Fig. 5b). For 2% w/v silk, the use of only the smallest gauge needle (30G) resulted in a substantial drop in cell viability when the gelled samples, rather than the liquid samples, were injected. This difference was less noticeable for 25G needles and was not observed for 21G needles. For 3% w/v silk samples, no difference was noted between the samples when a 21G needle was used. However, a significant and size-dependent reduction in cell viability was noted between gelled and solution samples after injection though 25G and 30G needles. The 4% w/v silk samples gave similar results, but a significant reduction in cell viability was even observed with a 21G needle (Fig. 5b). Following injection (i.e. needle shearing forces), cell viability was better preserved for all solution silk samples than for the respective hydrogels.

**Figure 6:**
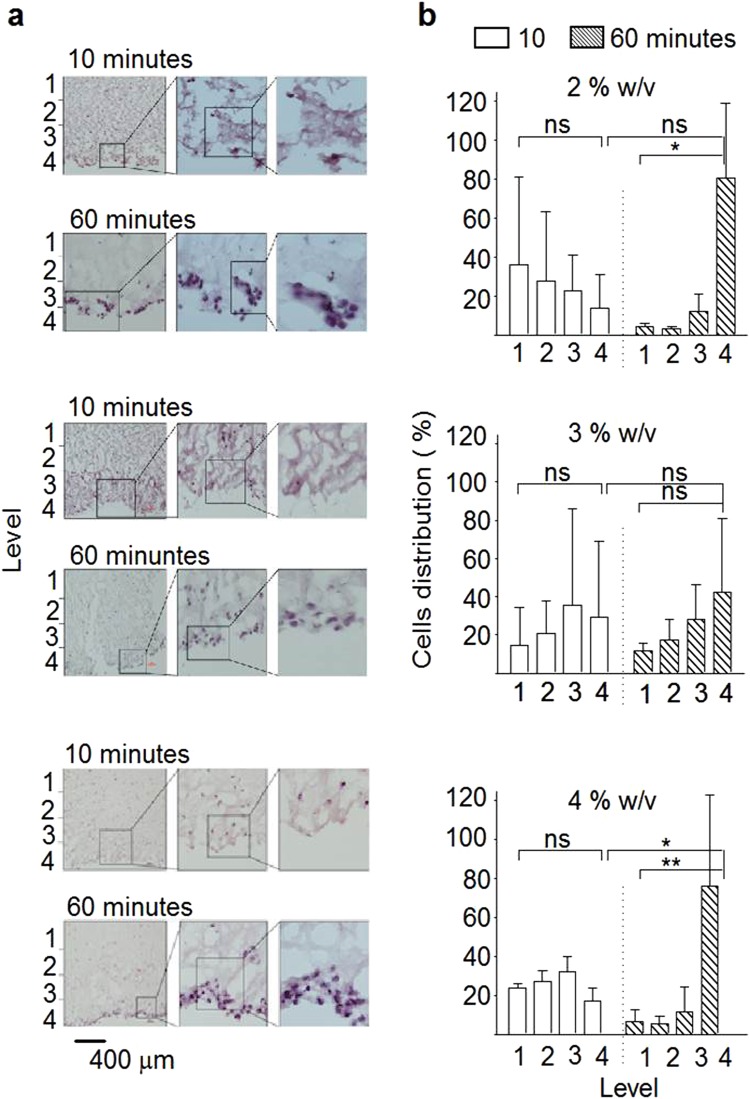
Impact of the solution-gel transition on cell distribution in self-assembling silk hydrogels *in vitro*. Cells were added at 10 minutes or 60 minutes before the completion of the solution-gel transition. (**a**) Histology images of MSCs encapsulated in silk hydrogels stained *in vitro* with H&E. Images were segmented into 4 regions (see Supplementary Fig. S4 for clarity of our methodological approach) and (**b**) the cell distribution was quantified. Note that for the 60 minute samples most of the cells had descended by gravity to the bottom of the hydrogel (level 4) (b, mean ± SD, n = 3 independent experiments; *P < 0.05, **P < 0.01).

Using an injectable biomaterial for cell delivery requires control over the (even) distribution of viable cells throughout the carrier matrix. Therefore, MSC distribution in the silk hydrogels was assessed by histology (Fig. 6a). MSCs were added to sonicated silk within 10 and 60 minutes of the solution-gel transition: at 10 minutes, an even distribution of cells was observed at every level throughout the hydrogel, whereas at 60 minutes, most cells had sunk to the bottom of the hydrogel due to gravity (Fig. 6b). Preliminary experiments in ischemic mice showed that addition of cells to silk undergoing the solution-gel transition within the 10 minute time window resulted in uniform cell distribution *in vivo* (Supplementary Fig. S2).

## Discussion

To date, no hydrogel system has been introduced into clinical practice for the delivery of cells to the brain, despite the position of several biopolymers at the clinical forefront. For example, multivalent cation-induced self-assembly of alginate has been exploited for encapsulation of genetically modified MSCs to express glucagon-like peptide-1 (i.e. GLP-1 CellBeads)^34^. These GLP-1 CellBeads have progressed to Phase I clinical trials (ClinicalTrials.Gov identifier: NCT01298830) for treatment of ischemic stroke. However, the lack of long-term mechanical robustness and challenges in navigating regulatory hurdles remain significant weaknesses of this delivery system. In addition, it ultimately requires surgical removal. Silk, by contrast, has a robust clinical track record in humans^24^ and our ability to reverse engineer silk is introducing new silk formats for novel applications^29^.

Cell delivery to the brain is emerging as a promising therapeutic strategy. However, current delivery technologies (e.g. simple injection of cells without a matrix) fall short of our insight into (stem) cell niches thus potentially undermining their therapeutic potential by stripping them of their supportive microenvironment (reviewed in^9,10^). We therefore explored the suitability of self-assembling silk hydrogels for future minimally invasive cell application; one potential scenario is the administration to the brain and for supporting stem cells. For minimally invasive brain administration, the solution-gel characteristics and substrate elasticity are critical so we therefore focussed on optimising the physical and chemical cues of self-assembling silk hydrogels for brain administration and stem cell support. Energy input into a silk solution displaces solvating water molecules and exposes the hydrophobic silk domains, which subsequently rearrange and grow into nanocrystallites^33^. Secondary structure analysis by FTIR and CD (Fig. 2b and c, respectively) showed that these nanocrystallites were β-sheets and thus represented the points of physical crosslinking within the hydrogel. These nanocrystallites are responsible for the observed light scattering and gave rise to the observed opaqueness of the silk hydrogels (this contrasts with chemically cross-linked silk hydrogels, which are transparent to visible light)^29^. As expected, the overall solution-gel kinetics were concentration dependent, with higher silk concentrations forming mechanically stable hydrogels more rapidly. The lower threshold for the formation of silk hydrogels was 1% w/v (Fig. 1d); in all probability, this concentration did not contain sufficient silk to form a mechanically robust hydrogel network, despite the formation of nanocrystallites. Overall, the observed changes in secondary structure and the solution-gel transition were in good agreement with those reported in previous studies^33,35^.

Control over mechanical properties, such as stiffness, is also essential to recreate an orphan tissue microenvironment that can support (stem) cell function, tissue growth and seamless integration with existing tissues^7^. Increasing silk content resulted in increased stiffness in the respective silk hydrogels due to the presence of nanocrystallites that provided mechanical strength (Fig. 1c). At 2 to 4% w/v, the silk hydrogels exhibited matrix elasticity similar to that of brain tissue (0.1 to 1 kPa, indicated by dotted line and shading, Fig. 1c)^36^. Qualitative assessment of these hydrogels by scanning electron microscopy showed a porous three-dimensional structure.

For the clinical translation of (self-assembling) hydrogels for intracerebral injection, good space-conforming properties without swelling are necessary to avoid compression against adjacent tissues, as this can further exacerbate cellular injury and clinical symptoms^9,10^. The self-assembling silk hydrogels exhibited no swelling during the solution-gel transition and showed excellent space conformity *in vitro* when injected in the pre-gelled state, especially at a higher silk content (Fig. 3a,b and c). In support of our *in vitro* results, preliminary *in vivo* experiments allowed visualisation of good space conformity in an ischemic cavity, in mice (Supplementary Fig. S1) and rats (Fig. 4) using 3% w/v and 4% w/v self-assembling silk hydrogels, respectively with no apparent adverse effects on animal health or behaviour (Supplementary Fig. S1). This observation agreed with the work by Fernández-García and co-workers, who showed that the use of silk hydrogels in healthy mice caused no considerable cognitive or sensorimotor deficits in either behavioural tests or electrophysiological analyses^37^ and, though outwith the scope of the present study, provide a platform for future experiments that characterise cellular phenotype and survival as well as compatibility in the brain tissue after transplantation. The ability to fill irregularly shaped defects by injecting a self-assembling material without the need for premoulding is essential for many therapeutic applications, including the stroke setting.

One therapeutic strategy is the use of MSCs (reviewed in^1,2,38^). Therefore, we examined the performance of MSCs following their embedding within self-assembling silk hydrogels *in vitro*. Over the course of the study period, the MSCs proliferated, although they showed a transient decrease in cell viability for the first 3 days (Fig. 5a). Similar observations have been made previously with (silk) hydrogel systems (e.g^33,39,40^.). For example, Wang and co-workers showed MSC proliferation over 21 days in 4% w/v silk hydrogels but negligible proliferation (and a degree of cell death) in 8% w/v and 12% w/v hydrogels. This effect of high silk concentrations likely reflects nutrient mass transport limitations and possible mechanical restrictions imposed by the hydrogels^33^.

We speculate that, over time, MSCs are able to modify the silk hydrogel by endogenous ECM deposition. The differential response of MSCs to 2 and 4% w/v silk hydrogels reported here suggests a direct influence of mechanobiology on MSC cell proliferation, which is not a unique effect of silk. For example, MSCs in collagen mimetic peptide hydrogels had better cell viability on softer hydrogels^40^; similar observations have also been made with different stem/progenitor cells and hydrogel systems (e.g. neural stem/progenitor cells and methacrylamide chitosan)^39^. MSC cultures growing on two-dimensional silk hydrogels showed a more spindle shaped morphology on soft silk hydrogels (6 kPa) and progressively spread out on stiffer substrates^41^. Treatment of MSCs with transforming growth factor 1 enabled the transition to a vascular smooth muscle cell phenotype and the best results were achieved using silk hydrogels of medium stiffness (33 kPa)^41^. Overall, (silk) mechanics is emerging as an important factor in MSC biological responses.

Application of a therapeutic payload to the brain is an appealing therapeutic avenue, but it requires minimally invasive administration of the payload. Delivery of MSCs requires a sustained cell viability and efficient cell distribution. We found that MSCs encapsulated in silk hydrogel that gelled within 10 minutes showed excellent cell distribution throughout the hydrogel, when compared to longer solution-gel transition times where gravity resulted in MSC settling (Fig. 6). Shear stress also affected MSC viability (Fig. 5b); as expected, cell viability was higher following injection from 21G needles than from narrower bore 25G and 30G needles, and the best results were achieved with pre-gelled systems. In human clinical trials, delivery of stem cells into the cavity typically requires a custom-made needle with an internal diameter of 0.35 mm (similar to 22G to 23G) (e.g.^42^). We therefore used gauge size 22G and 26G needles in our rat and mouse pre-clinical stroke models, respectively. These needle sizes limit tissue damage but are also relevant for cell delivery. Preliminary studies in mouse stroke model showed uniform cell distribution (Supplementary Fig. S2).

## Conclusions

Taken together, this study provides valuable information regarding the optimal MSC-hydrogel matrix combinations for future experimental studies targeting central nervous system repair. The key findings of the present study are that (i) self-assembling silk hydrogels exhibit controllable solution-gel kinetics, elasticity, and secondary structure, (ii) these self-assembling silk hydrogels can be fine-tuned to achieve uniform cell distribution, viability and space conformity in the absence of any silk hydrogel swelling; and (iii) preliminary proof-of-concept studies allowed visualisation of space conformity of self-assembling silk hydrogels after injection into the brain. Overall, our *in vitro* data indicates that 3% w/v silk hydrogels, which gelled within 10 min of MSC addition, should be injected in the pre-gelled state to provide optimal support of MSC viability and distribution.

## Materials and Methods

### Silk solution preparation

*Bombyx mori* silk cocoons were reverse engineered as detailed previously^28^; for a video-based protocol, see^43^. Briefly, dried cocoons (Tajima Shoji Co., Yokohama, Japan) were degummed for 60 minutes in 25 mM Na_2_CO_3_ and then rinsed in ddH_2_O to remove sericin. Dried degummed silk fibres were dissolved in 9.3 M LiBr solution at 60 °C for 3 to 4 hours. The resulting solution was dialysed against ddH_2_O using a dialysis cassette (molecular cut off of 3,500 Da; Thermo Fisher Scientific Inc., Waltham, MA, USA) for 2 days, with several changes of water, to remove LiBr salts. The resulting aqueous silk solution (typically between 6 to 7% w/v) was centrifuged to remove any silk aggregates and the resulting silk solution was stored at 4 °C until use.

### Manufacture, monitoring and characterisation of self-assembling silk hydrogels

Self-assembling silk hydrogels were prepared using a digitally controlled probe sonicator (Sonoplus HD 2070, Bandelin, Berlin, Germany) fitted with a 23 cm long sonication tip (0.3 cm diameter tip and tapered over 8 cm). Unless otherwise stated, 4 to 8 ml sample batches in 15 ml Falcon tubes (1.4 cm diameter and 11 cm long) (Greiner Bio-One GmbH, Kremsmünster, Austria) were exposed to a 30% amplitude for typically 3 to 6 sonication cycles on ice (one cycle consisted of 30 seconds on and 30 seconds off) to induce the solution-gel transition of 1 to 5% w/v silk samples (Fig. 1a). One method used to track the solution-gel transition was to measure light scattering at 405 nm (Spectra Max M5, Molecular devices, Sunnyvale, CA, USA), because physically crosslinked silk hydrogels develop nanocrystalline regions that scatter light^29^. In the present study, this was done by subjecting the silk solution samples to two 30 second sonication cycles, and light scattering was monitored for 24 hours.

Circular dichroism (CD) spectroscopy was used to assess the kinetic transition from a random coil to a stable β-sheet. Following sonication, samples were immediately loaded into a 0.01 mm path length measuring cell. The temperature was kept constant at 25 °C, and the samples were scanned at one single time with a CD spectrometer (Chirascan-plus, Applied Photophysics Ltd, Leatherhead, UK) over the 190 to 250 nm spectrum with a 1 nm resolution and 3 scans per time point over 24 hours. The change in ellipticity at 217 nm was used to monitor β-sheet formation. The silk secondary structure was assessed using Fourier transform infrared (FTIR) spectroscopy, with measurements being repeated (total twice). For FTIR measurements, the silk hydrogel samples were exposed to 128 scans at 4 cm^−1^ resolutions over the wavenumber range 400 to 4000 cm^−1^ using a TENSOR II FTIR spectrometer (Bruker Optik GmbH, Ettlingen, Germany). Baseline and peak fit were corrected by OriginPro 9.2 software at the amide I region (1595–1705) as detailed previously^44^. The amide I region was identified and deconvoluted, as follows: 1605−1615 cm^−1^ as side chain/aggregated strands, 1616−1637 cm^−1^ and 1697−1703 cm^−1^ as β-sheet structure, 1638−1655 cm^−1^ as random coil structure, 1656−1662 cm^−1^ as α-helical bands and 1663−1696 cm^−1^ as β-turns.

For rheological assessments, 2 to 5% w/v silk hydrogels (0.2 ml) were prepared and equilibrated in PBS overnight. The hydrogels were then subjected to rheological characterization (Kinexus pro+ rheometer, Malvern Instruments Ltd., UK) using a 20 mm diameter plate set to 25 °C and appropriate gap size. Strain stress was measured first from 0.1 to 100% with at a set frequency of 1.0 Hz, followed by a frequency sweep.

For scanning electron microscopy (SEM), silk hydrogels were frozen and then freeze dried (Micro Modulyo, Thermo Fisher Scientific Inc.). Dried samples were immediately moved to a desiccator and sputter coated with carbon using a vacuum coater (Polaron Division E6100, Bio-Rad, Birmingham, UK). Coated samples were imaged twice with a FE-SEM SU6600 scanning electron microscope (Hitachi High Technologies, Krefeld, Germany) using 5 kV acceleration voltage. Electron micrographs were analysed using ImageJ v1.50i (National Institutes of Health, Bethesda, MD, USA).

### Assessment of silk hydrogel swelling and space conformity

To determine silk hydrogel swelling, 300 µl of sonicated silk solution was added to pre-weighed Eppendorf tubes and weighed. Once the hydrogel had gelled, phosphate buffered saline (PBS; 300 µl) was added carefully on top of the silk hydrogel, the tubes were re-weighed and left for 24 hours. After complete removal of PBS, the final weight was determined. The swelling ratio was calculated using the initial silk solution weight and the final weight after 24 hours of PBS incubation. The silk hydrogels were generated (detailed above) and subjected to *in vitro* space conformity testing. Sonicated silk samples were drawn up into 1 ml syringes (without a needle), and either (i) the silk sample was left in the syringe to complete the solution-gel transition and subsequently transferred to a cuvette, or (ii) the silk sample was immediately transferred from the syringe to the cuvette (i.e. still in its liquid form) and allowed to gel in the cuvette. In both conditions, the cuvettes were filled with the silk hydrogel samples up to the 1 ml mark. The cuvettes were then weighed and the conformity was calculated using the theoretical weight of the respective 1 ml sample as 100%.

### Preparation and assessment of mesenchymal stem cells in self-assembling silk hydrogels

Mouse C3H10T0.5 mesenchymal stem cells (American Type Culture Collection) were grown on tissue culture treated polystyrene flasks (Corning Inc., New York, USA) and cultured in Roswell Park Memorial Institute medium 1640 containing 4.5G GlutaMAX, supplemented with 10% v/v foetal bovine serum and penicillin and streptomycin (50 U/mL penicillin and 50 μg/mL streptomycin) (Thermo Fisher Scientific). Cells were maintained in a humidified atmosphere of 5% CO_2_ at 37 °C, and subconfluent cultures were routinely subcultured with trypsin/EDTA every 2 to 3 days.

For silk hydrogel cell culture studies, the silk solution was filter sterilised (33 mm Millex-GP syringe filter fitted with a polyethersulfone membrane with 0.22 µm pores) and the silk solution was sonicated on ice in a class II biological safety cabinet. Next, 10× PBS was added to the aqueous silk solutions to yield an isotonic solution and C3H10T0.5 cells (2 × 10^5^ cells per 50 μl of silk were added to the processed silk samples at 28 °C to 32 °C). The exact timing of cell addition was established by monitoring the flow characteristics of the silk sample. Uniform cell seeding throughout the silk hydrogel was achieved by adding cells once the initiation of the solution-gel transition was evident. The MSC silk mixture (2 × 10^5^ cells in 50 μl) was pipetted into a 96-well plate well (well surface area 0.32 cm^2^) and transferred to the cell incubator for 10 to 30 minutes to allow completion of the solution-gel transition. Next, 200 µl of complete culture medium was carefully added on the top of the mixture, and this was changed every 3 days. Cell viability was assessed by adding 25 μl AlamarBlue (Thermo Fisher Scientific) to the respective well and the cells were allowed to metabolise the substrate for 4 hours. The culture supernatant was then transferred to a black 96-well plate (Sigma-Aldrich, St. Louis, MO, USA) and measured with a fluorescence plate reader (POLARstar Omega BMG LABTECH GmbH, Ortenburg, Germany) by fixing the photo multiplier tube and setting the excitation and emission filters to 560 nm and 590 nm, respectively.

The viability of the MSCs in silk hydrogels post-injection was assessed by injecting 50 µl of silk MSC sample mixtures in either the pre-gelled (i.e. in the liquid form) or post-gelled state through different needle gauges (21, 25 and 30G) into 96-well plates to establish the effect of shear stress on cell viability. The cell viability was assessed within 180 minutes after injection using AlamarBlue, as detailed above. This short time frame was selected to assess shear stress induced changes in viability while minimizing differences due to proliferation.

In a final set of *in vitro* studies, the distribution of encapsulated MSCs in silk hydrogels was assessed by histology. The solution-gel transition was fine tuned to occur within 10 or 60 minutes. During this solution-gel transition time window, cells were added to the silk samples and the MSC silk mixture was transferred into 24-well plate Transwell inserts with the smallest pore size of 0.4 µm to mitigate sample leakage (Corning Life Sciences B.V., Amsterdam, The Netherlands). These Transwell inserts have a surface area of 0.32 cm^2^ and therefore resemble the 96-well plate format used in our study. To facilitate sample handling, the MSC silk mixture was scaled up to 200 µl. Once the silk samples had completed their solution-gel transition, the Transwell membrane was cut to facilitate removal of the intact silk hydrogels. The hydrogels were fixed in 4% paraformaldehyde for 30 minutes, then immersed in sucrose (30% w/v) overnight at 4 °C, frozen in isopentane/dry ice at −42 °C for 2 minutes and cryosectioned. The 20 µm sections were stained using haematoxylin and eosin. A minimum of 3 consecutive sections per sample were analysed. Each image was segmented into 4 equal levels, based on their depth in the hydrogel in the Transwell, with level 4 being at the bottom of the hydrogel in the Transwell and level 1 being at the top of the hydrogel in the Transwell (see Supplementary Fig. S4). Levels 1–4 are shown on the Fig. 6a on the left hand sides of the images. The number of cells in each level was counted. The purpose of the 4 levels was to quantify whether the cells had sunk to the bottom of the hydrogel in the Transwell or whether the cells had equal distribution throughout the hydrogel.

### Rat stroke model and administration of self-assembling silk hydrogels

Preliminary experiments were designed to allow visualisation of space conformity *in vivo*. All *in vivo* studies were approved by the Home Office of the United Kingdom (Project License Number 60/4469). All procedures complied with the UK Animals (Scientific) Procedures Act (1986) and the Ethical Review Process of the Institute of Pharmacy and Biomedical Sciences at the University of Strathclyde. The *in vivo* studies followed the ARRIVE guidelines^45^. Animals were kept under a 12 hour light/dark cycle and were fed *ad libitum*. The *in vivo* studies were conducted in rodent models. The rat data are shown in the manuscript. The mice data are shown in Supplementary information only.

The rat study was performed as detailed previously^46^. Briefly, male Sprague-Dawley rats (n = 4, weight 240–290G, 8–9 weeks, Harlan, UK) underwent a right transient middle cerebral artery occlusion for 60 minutes by insertion of a propylene filament (Doccol Corporation; tip diameter with coating 0.33 +/− 0.02 mm), via the common carotid artery to the ostium of the middle cerebral artery in the circle of Willis. A priori exclusion criterion was any animal found to be moribund due to excessive weight loss (>20% of start weight). No animals were excluded based on this exclusion criteria, however one animal was excluded due to damage to tissue during processing of the tissue for histology, leading to a final n number n = 3. At 14 days after this middle cerebral artery occlusion, 10 μl of 4% w/v self-assembling silk hydrogel was injected at a rate of 2 μl/minute at coordinates (L) −1.5 mm, (A-P) −3.5 mm and (V) −6.5 mm, using a Hamilton syringe with a 22 gauge blunt tip needle. Animals were terminally anaesthetised at 1 week after the transplantation by overdosing with sodium pentobarbital. A transcardial perfusion of 0.9% w/v saline was followed by 4% w/v ice-cold paraformaldehyde in 0.2 M PBS. Brains were removed following craniotomy and were fixed in paraformaldehyde for 24 hours. The brains were then immersed in cryoprotective solution (30% w/v sucrose in PBS with 0.01% w/v sodium azide for 72 hours, followed by rapid freezing on dry ice). Coronal (40 μm) cryostat sections were cut and stained with haematoxylin and eosin.

### Statistical analyses

Data were analysed using GraphPad Prism 7.0 (GraphPad Software, La Jolla, CA, U.S.A.). Sample pairs were analysed with the Student’s t-test. Multiple samples were evaluated by one-way analysis of variance (ANOVA), followed by Dunnett’s multiple comparison post hoc test. Asterisks were used to denote statistical significance as follows: *P < 0.05, **P < 0.01, ***P < 0.001. All data were presented as mean values ± standard deviation (SD). The number of independent experiments (n) is noted in each figure legend.

## Electronic supplementary material

Supplementary Information

## Data Availability

All data created during this research are openly available from the University of Strathclyde-Pure, at 10.15129/2bbce19a-4b47-43d9-b9d5-7549b4d9ae4a.
