# Supplementary material for: In vitro studies on space-conforming self-assembling silk hydrogels as a mesenchymal stem cell-support matrix suitable for minimally invasive brain application
Source: Sci Rep. 2018 Sep 12;8:13655. doi: 10.1038/s41598-018-31905-5 (PMC6135807; doi:10.1038/s41598-018-31905-5)
Supplement: Supplementary file 1 — Supplementary Information [file 41598_2018_31905_MOESM1_ESM.docx]

***In vitro* studies on space-conforming self-assembling silk hydrogels as a mesenchymal stem cell-support matrix suitable for minimally invasive brain application.**

Osama I.^1^, Gorenkova, N.^1^, McKittrick, C.M.^1^, Wongpinyochit, T.^1^, Goudie, A.^1,^  Seib, F.P.^1,2^*^ƚ^, Carswell, H.V.O.^1^*^ƚ^

^1^Strathclyde Institute of Pharmacy and Biomedical Sciences, University of Strathclyde, Glasgow, UK; ^2^Leibniz Institute of Polymer Research Dresden, Max Bergmann Center of Biomaterials Dresden, Hohe Strasse 6, 01069 Dresden, Germany

**Supplementary Information**

*Mouse stroke model and administration of self-assembling silk hydrogels*

Male C57BL/6 mice, aged 10–12 weeks, underwent cerebral ischemia by left middle cerebral artery occlusion by either permanent occlusion by electrocoagulation (pMCAO)^50^ (n=4) (for Fig S1) or by transient occlusion by intraluminal thread for 45 minutes (tMCAO)^51^ (n=6) (for Fig S2). All mice were anaesthetised with 3% isoflurane (Bimeda MTC Animal Health Inc., Dublin, Ireland) mixed with 1% oxygen and maintained with 1.5 ± 0.25% isoflurane. The core body temperature was regulated at 37 ± 0.5 °C. A priori exclusion criterion was any animal found to be moribund due to excessive weight loss (> 20% of start weight). No mice were excluded from the present study.

At 2-4 months post-pMCAO, mice received self-assembling silk hydrogel implants either by topical application or by intracerebral injection. Silk hydrogels were prepared analogous to the *in vitro* studies. By topical application, 3 % w/v silk hydrogel was used (Fig.S1a). Mice (n=2) were euthanised, placed on a heat pad to maintain the temperature at 37 °C, and an incision was made to expose the skull. A craniectomy was then performed to expose the stroke cavity. The 3% w/v silk hydrogel was sonicated, and before completion of the solution-gel transition, 20 ul was injected onto the surface of the cavity and left for 1 hour to ensure complete gelation. The brain was then removed and processed for haematoxylin and eosin staining.

By intracerebral injection, 4 % w/v silk hydrogel was used (Fig S1b). Mice (n=2) were anaesthetised with isoflurane and mounted on a stereotaxic frame. Using Bregma as a reference point, a Hamilton syringe was moved to the following coordinates (medial/lateral +2.5 mm, anterior/posterior -0.5 mm) to drain the ECF and the second burr hole 1 mm caudally as previously described^52^ to prevent increase in intracranial pressure with biomaterial injection. A 26 gauge needle was inserted 1.5 mm ventrally, 5ul of 4 % w/v silk hydrogel was injected over 2 minutes, and the syringe was left in place for another 5 minutes. Mice were sutured and allowed to recover for additional 10 mins before termination. The brain was then removed and processed for haematoxylin and eosin staining.

Silk hydrogels for cell delivery were performed analogous to the *in vitro* studies (Fig S2). CellTracker CM-DiI (Thermo Fisher Scientific) labelled cells were added to self-assembling silk hydrogels within the last 10 minutes of the solution-gel transition time window and injection. At 3 days post-tMCAO mice, mice were randomly assigned to receive either CellTracker CM-DiI-labelled C3H10T0.5 cells (2 × 10^4^ cells/5 μl) embedded in 3 % w/v self assembling silk hydrogels (n=3) or C3H10T0.5 cells (2 × 10^4^ cells/5 μl) with no hydrogel (n=3) by stereotaxic injection (medial/lateral +1.5mm, anterior/posterior +1 mm, -2.5 mm ventral relative to bregma). Mice were allowed to recover for 3 hours before termination. The brain was then removed and processed for haematoxylin and eosin staining.

**References**

50. Fumagalli, S, Coles, J.A.C., Ejlerskov, P., Ortolano, F., Bushell, T.J., Brewer, J.M., De Simoni, M.G., Dever, G., Garside, P., Maffia, P., Carswell, H.V *In vivo* Real Time Multiphoton Imaging of T Lymphocytes in Mouse Brain After Experimental Stroke*. Stroke* **42**; 1429-1436 (2011).

51. McKittrick, C. M., Lawrence, C. E. & Carswell, H. V. Mast cells promote blood brain barrier breakdown and neutrophil infiltration in a mouse model of focal cerebral ischemia. *J. Cereb. Blood Flow Metab.* **35**, 638-647, (2015).

52. Massensini, A.R., Ghuman, H., Saldin, L.T., Medberry, C.J., Keane, T.J., Nicholls, F.J. et al., Concentration-dependent rheological properties of ECM hydrogel for intracerebral delivery to a stroke cavity, Acta Biomater. **27;** 116e130, (2015)

**
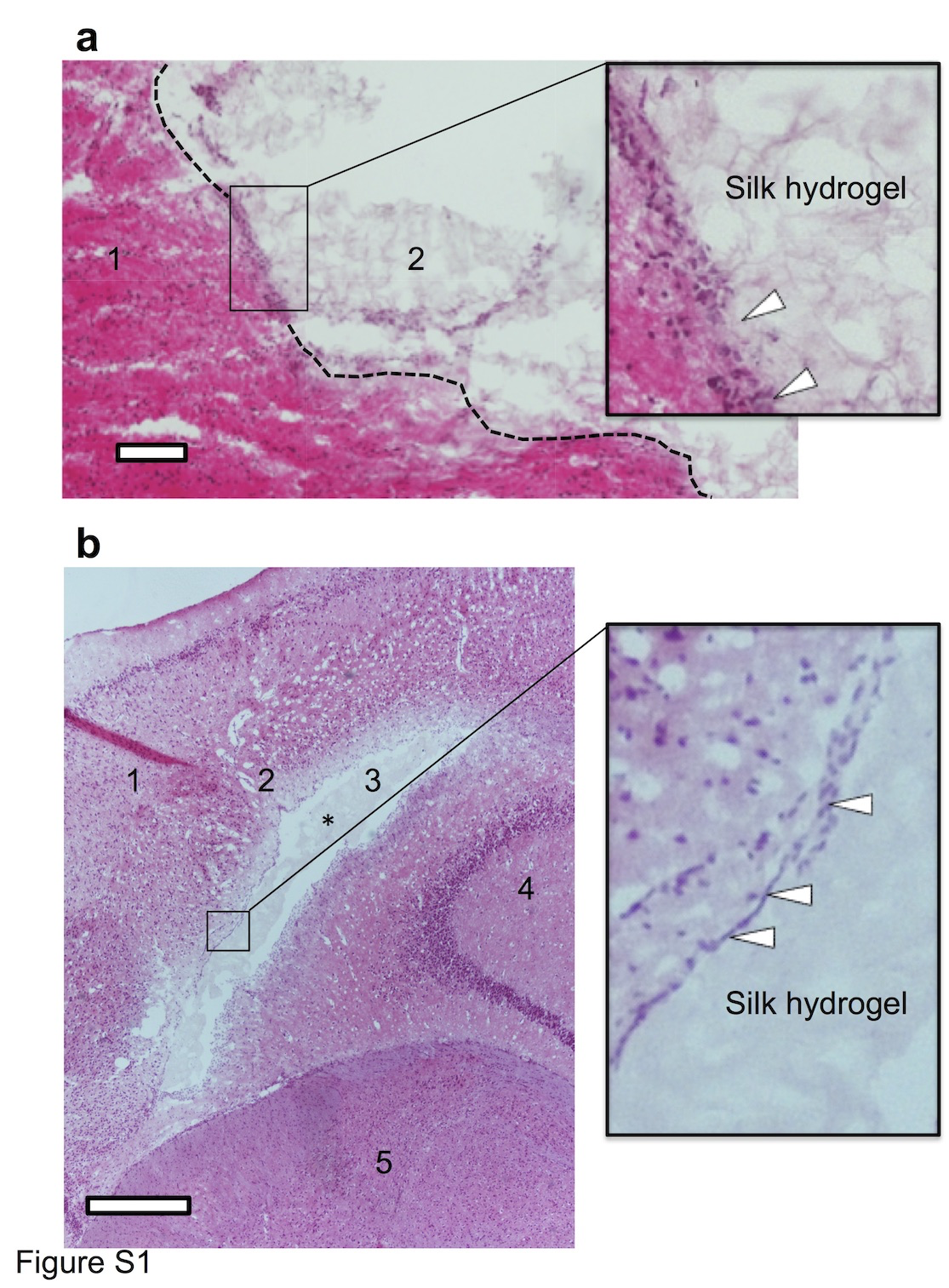
**

**Supplementary Figure S1.** Representative H&E stained sections showing space conformity of self-assembling silk hydrogels in the mouse brain after cerebral ischemia. (a) 3% w/v self-assembling silk hydrogel applied topically after pMCAO at the level of the lateral habenula (interaural distance 1.98 mm). 1: brain tissue, dotted line: brain edge of stroke cavity; 2: silk hydrogel. Arrows tissue-silk hydrogel interface. Scale bar 100 μm. (b) 4 % w/v self-assembling silk hydrogel injected intracerebrally after pMCAO showing that self-assembling silk hydrogel accumulated in the lateral ventricle cavity. 1: viable cortical tissue; 2: border of viable tissue and infarct; 3: silk hydrogel; 4: viable hippocampal tissue. Arrows tissue-silk interface. Scale bar 500 μm.

**
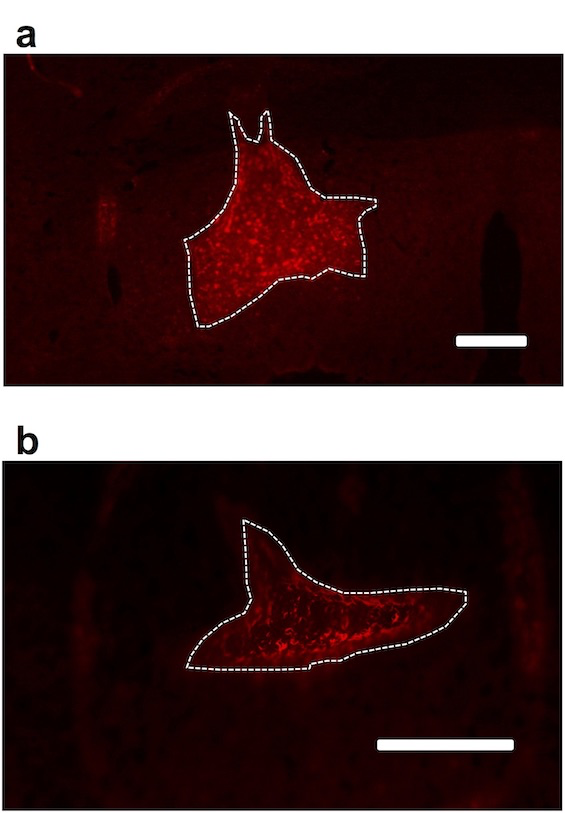
**

**Supplementary Figure S2.** Representative fluorescent images of space conformity and distribution of CellTracker CM-DiI labelled MSCs delivered to the mouse brain after cerebral ischemia using self-assembling silk hydrogels within the MCA territory at the coronal levels of (a) the septal nucleus (interaural distance 3.94mm) and (b) nucleus accumbens (interaural distance 4.66mm). Dotted line outlines silk hydrogel, scale bars (a) 100 μm and (b) 200 μm.


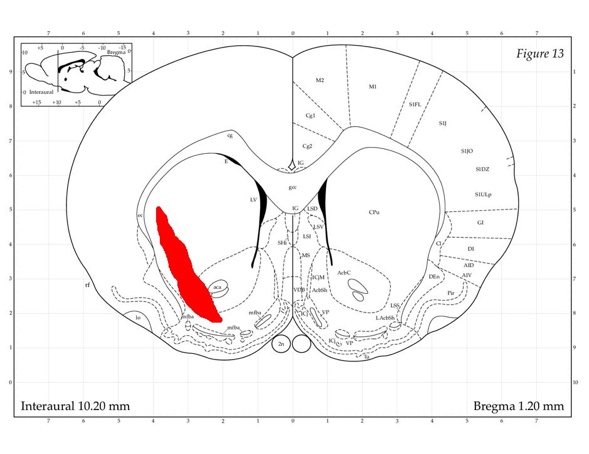

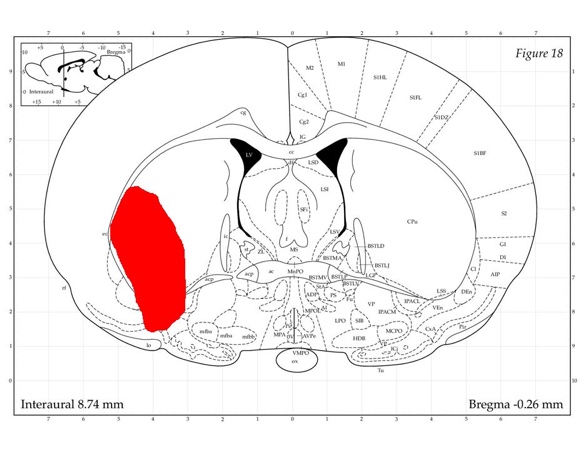
**a b**

**Supplementary Figure S3.** Representative line diagrams showing the injury topography in rats injected with silk hydrogel graft 2 weeks after onset of cerebral ischemia. (Box indicates the lesion with silk hydrogel graft area with H&E staining, shown on Fig 4 b,c.) **a**,- rat brain section with small striatal lesion, Bregma AP=1.20. **b**,- rat brain section with striatal lesion, Bregma AP=-0.26.

**(Atlas Source:** Paxinos, George, and Charles Watson. *The rat brain in stereotaxic coordinates: hard cover edition.* Access Online via Elsevier, 2006.)


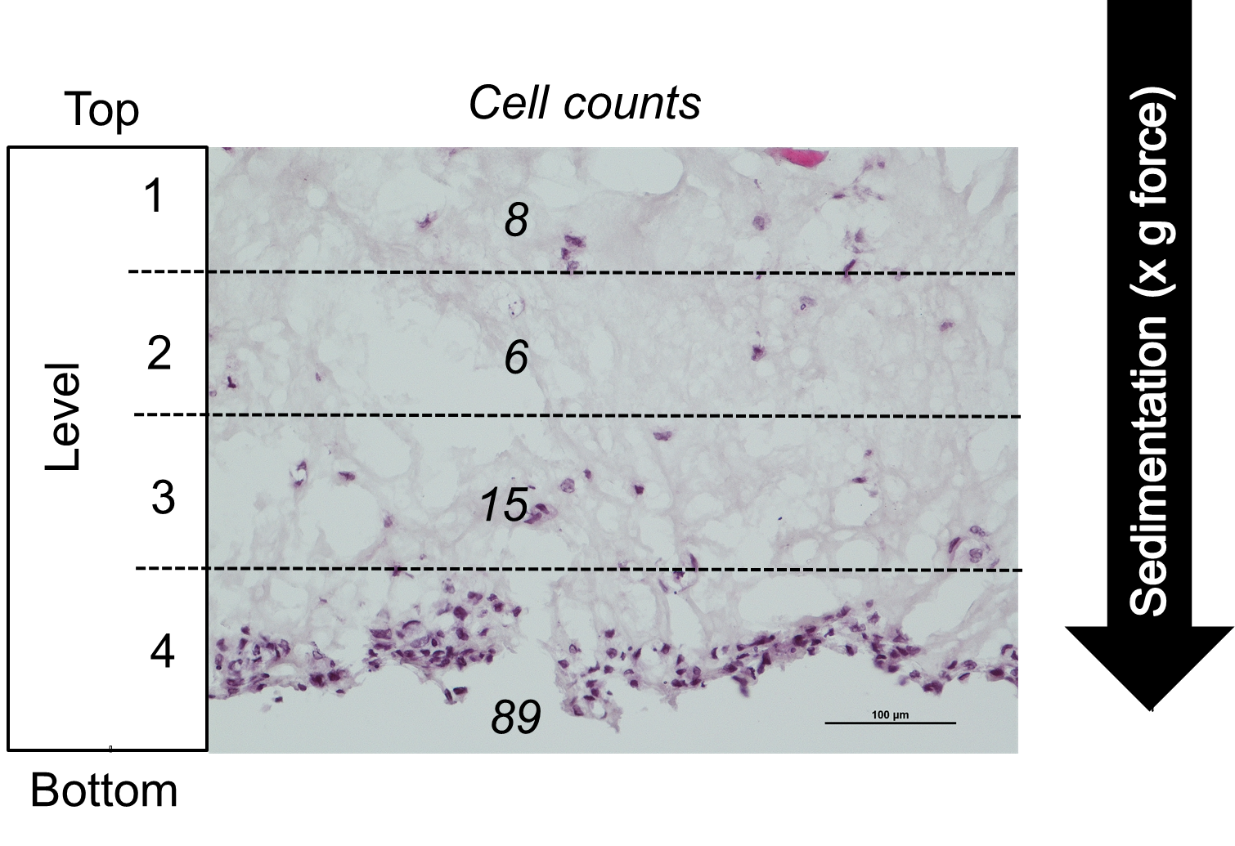


**Supplementary Figure S4.** Representative histology image of MSCs encapsulated in silk hydrogels stained *in vitro* with H&E, illustrating how each image was segmented into 4 equal levels, based on their depth in the hydrogel in the Transwell (level 4 at the ‘bottom’ of the hydrogel in the Transwell and level 1 at the ‘top’) and illustrating the number of cells at each level for that particular image indicating that the cells had sunk to the bottom of the hydrogel rather than exhibiting an equal distribution throughout the hydrogel.
